# Vivisection Questioned
*"The Healing Art and the Claims of Vivisection." By Edward Berdoe, M.R.C.S. (Eng.), L.R.C.P. (Edin.). (London: Swan Sonnenschein and Co., Paternoster-square.) Price one shilling.


**Published:** 1891-05-16

**Authors:** 


### VIVISECTION QUESTIONED.*

Of all the opponents of vivisection, none is more earnest than Mr. Edward Berdoe, and though we may think that "Æsculapius Scalpel" sometimes uses his knife unfairly, it is undeniable that he has brought a strong case against experiments on living animals in the lecture which he delivered at Cambridge a year ago, and which he has now published in a slender volume, under the somewhat sarcastic title of "The Healing Art and the Claims of Vivisection." His contention is, in brief, that the art of healing has gained nothing by vivisection; that all the sufferings of dogs, cats, rabbits, and the rest have not saved humanity a single pang. Science in the abstract may have profited, but the British public, while sufficiently selfish to let every animal under the sun be experimented on for their own comfort, would not sacrifice a single mouse to science in the abstract. People tolerate vivisection in the belief that these experiments made *in corpore vile* save the higher human animal some pain; if Mr. Berdoe can prove that no one gains by all this suffering except some morbidly curious scientists the knell of vivisection has begun to sound.

Certainly he brings forward a strong case. To begin with, he narrates some of the experiments that have been made in recent times, and the list is sufficiently appalling. Then he goes on to show that though vivisection has occasionally demonstrated ascertained facts, it has never led to the discovery of any new ones. "To put a plaster-of-Paris jacket on a guinea-pig or rabbit," he says, "and so constrict its breathing and other organs as to kill it, is not to discover the evils of tight-lacing by experiments on living animals. These evils have all been recognised before, and the *post-mortem* table will unhappily continue to exhibit them abundantly."

But this is only a small part of the contention. Mr. Berdoe shows that the results of experiments as to the effect of drugs on the lower animals cannot be regarded as proving what will be their action on the human subject. Aconite may be eaten with impunity by horses, and, according to Dr. Stillé and Dr. Maisch, animals which have partaken of belladonna do not show "any degree of that delirious excitement which it produces in man." On the other hand, if substances that are poisonous to us have no effect on the lower animals, some things we count harmless are fatal to some of them. "Citric acid is a powerful poison to cats, rabbits, and other animals, causing violent convulsive spasms." "Common glycerine injected subcutaneously into dogs produces death, with symptoms of alcoholic poisoning." Had experiments on animals preceded the popular use of these and other drugs we certainly would have had none of them. It seems, then, that experiments on animals constituted differently from ourselves are often unreliable. Apart from the fact of this difference of constitution it must be remembered that we take the drug by the mouth, while with them it is injected beneath the skin or into the intestine; that we take it willingly, they under often cruel compulsion. These things create a different set of physiological circumstances which tend still further to confuse results.

Mr. Berdoe even brings the vivisectors themselves to bear witness on his side. He quotes Dr. Percy Wilde's remarks on some experiments with calomel: "They only prove that calomel given to dogs, in a certain dose, and under certain conditions, diminishes the secretion of bile; but as to its action upon the human body, either in health or disease, they prove nothing." M. Claude Bernard gives testimony that seems absolutely fatal to vivisection when he says: "Not only do the various species of animals differ in this respect, but even individuals of the same species are so far from resembling each other that they cannot be submitted to the same experiments." A greyhound will die under an operation that would lead to no result if tried on a sheep-dog. And which of these is the more akin to man? "An irritable, sensitive, and highly organised nervous system is, in fact," says Bernard, "the essential difference which separates a race-horse from one of those diminutive, half-wild ponies which hilly countries so abundantly produce. Would not the results of the same experiments be entirely at variance in these different animals?" And by which results should the physician go in dealing with a human patient? One can imagine a conscientious and practical vivisector who wants his labour to bear some fruit, tearing his hair at such utterances as these from a master of the craft. This pamphlet is, indeed, one of the most effective attacks that have been brought against the practice of vivisection, for it is founded not on sentiment, with which we may sympathise, but by which we will not guide our actions; but on facts—facts drawn mainly from the results of vivisection itself.

* "The Healing Art and the Claims of Vivisection." By Edward Berdoe, M.R.C.S. (Eng.), L.R.C.P. (Edin.). (London: Swan Sonnenschein and Co., Paternoster-square.) Price one shilling.

